# Malaria vector abundance is associated with house structures in Baringo County, Kenya

**DOI:** 10.1371/journal.pone.0198970

**Published:** 2018-06-11

**Authors:** Isabella M. Ondiba, Florence A. Oyieke, George O. Ong’amo, Macrae M. Olumula, Isaac K. Nyamongo, Benson B. A. Estambale

**Affiliations:** 1 School of Biological Sciences, University of Nairobi, Kenya; 2 Division of Research Innovation and Outreach, Jaramogi Oginga Odinga University of Science and Technology, Bondo, Kenya; 3 Cooperative Development, Research and Innovation, The Cooperative University of Kenya, Nairobi, Kenya; Swedish University of Agricultural Sciences, SWEDEN

## Abstract

Malaria, a major cause of morbidity and mortality, is the most prevalent vector borne disease in Baringo County; a region which has varied house designs in arid and semi-arid areas. This study investigated the association between house structures and indoor-malaria vector abundance in Baringo County. The density of malaria vectors in houses with open eaves was higher than that for houses with closed eaves. Grass thatched roof houses had higher density of malaria vectors than corrugated iron sheet roofs. Similarly, mud walled houses had higher vector density than other wall types. Houses in the riverine zone were significantly associated with malaria vector abundance (p<0.000) possibly due to more varied house structures. In Kamnarok village within riverine zone, a house made of grass thatched roof and mud wall but raised on stilts with domestic animals (sheep/goats) kept at the lower level had lower mosquito density (5.8 per collection) than ordinary houses made of same materials but at ground level (30.5 mosquitoes per collection), suggestive of a change in behavior of mosquito feeding and resting. House modifications such as screening of eaves, improvement of construction material and building stilted houses can be incorporated in the integrated vector management (IVM) strategy to complement insecticide treated bed nets and indoor residual spray to reduce indoor malaria vector density.

## Introduction

In order to achieve the World Health Organization strategy of eliminating malaria in 35 endemic countries, supplementary interventions among them improved housing [1,2], have been recommended. In Kenya, about 70% of the population is at risk of malaria with the burden being realized more in the riparian ecosystems[3]. The disease is seasonal in semi-arid areas but a recent study indicated that malaria is also perennial in low altitude areas of semi arid Baringo County[4]. Furthermore, malaria out breaks were experienced in the eastern part of Baringo in 2017 and over 20 lives were lost with the most affected being children. Though the cases were attributed to previous rainfall and shortage of mosquito nets, the question to be answered is whether environmental factors including house designs and structures in the county contributed to escalated malaria cases. An investigation conducted in western Kenya by Zhou *et al*. also found that house structure variables significantly affected abundance of indoor resting mosquitoes among other environmental factors[5].

*Anopheles gambiae* and *Anopheles funestus* which are the competent malaria vectors in Baringo County mainly bite and rest indoors. Consequently, one of the malaria control strategy is through protection against vectors using insecticide treated bed nets (ITNs) and indoor residual spray (IRS) interventions[6]. However, IRS operations have not been conducted in the affected parts of Baringo since malaria is seasonal and therefore national malaria control programs usually focus on endemic areas of the country[7]. Besides, residents affected by the recent malaria out break complained of lacking bed nets making it possible for their children to be exposed to mosquito bites at night. To confirm their concerns, studies have shown that exposure to mosquito bites still occurs indoors as the people spend most of the dark hours indoors when mosquitoes are active[8,9]. Additionally, most *Plasmodium*-infected mosquitoes are still found indoors (Okumu *et al*. unpublished data) therefore improved house-based interventions remain relevant for both malaria control and elimination.

Recent studies have shown that *An*. *gambiae* approaches the house at eave level and is attracted by carbon dioxide plume coming from the house[10]. Thus, there is need for additional measures to supplement ITNs and IRS to prevent house entry and human-vector contact. One of such a measure that has been tested under semi field conditions is use of eave tubes treated with insecticide[11–13]. Evidence from recent studies in Mbita, Kenya and Ikafara,Tanzania have shown that house modification by insertion of treated eave tubes can contribute significantly towards malaria control by optimizing insecticide delivery against mosquitoes[14,15]. Open eaves are sometimes inevitable, depending on climatic conditions of an area, and have been associated with malaria in children in Ethiopia [16] and Equatorial Guinea[17].

Housing structure has been shown to be one of the factors influencing indoor vector densities and malaria transmission. A number of studies conducted elsewhere have demonstrated an association between vector density and housing structure[17–20]. Evidence from a study in the Gambia shows that improved housing reduced the prevalence of anaemia in children but this intervention has been neglected for decades[21,22]. Arid and semi-arid areas of Baringo County [23] have varied house designs but a comprehensive study on malaria vector abundance with respect to house structures has not been undertaken. The County is inhabited by three main communities namely Tugen (dominant community), Ilchamus and Pokots. Each community has its own unique design of the houses leading to great diversity of housing structures. Therefore, an observational study to gather more evidence with the view of contributing to the growing body of knowledge on association between malaria and house structure was conducted in Baringo County. The findings can be relevant in the design of customized integrated vector control strategies in the heterogeneous topography and culture of Baringo County. A demographic survey in Kenya indicated that corrugated iron sheet roofed houses have been increasing from 1993 to 2009 while thatch roof has decreased[24]. The aim of this study was to assess the association of house structure on the abundance of indoor-resting malaria vector mosquitoes in selected study sites in Baringo County to ascertain link between malaria vectors and type of housing.

## Materials and methods

### Study area

**Fig 1:**
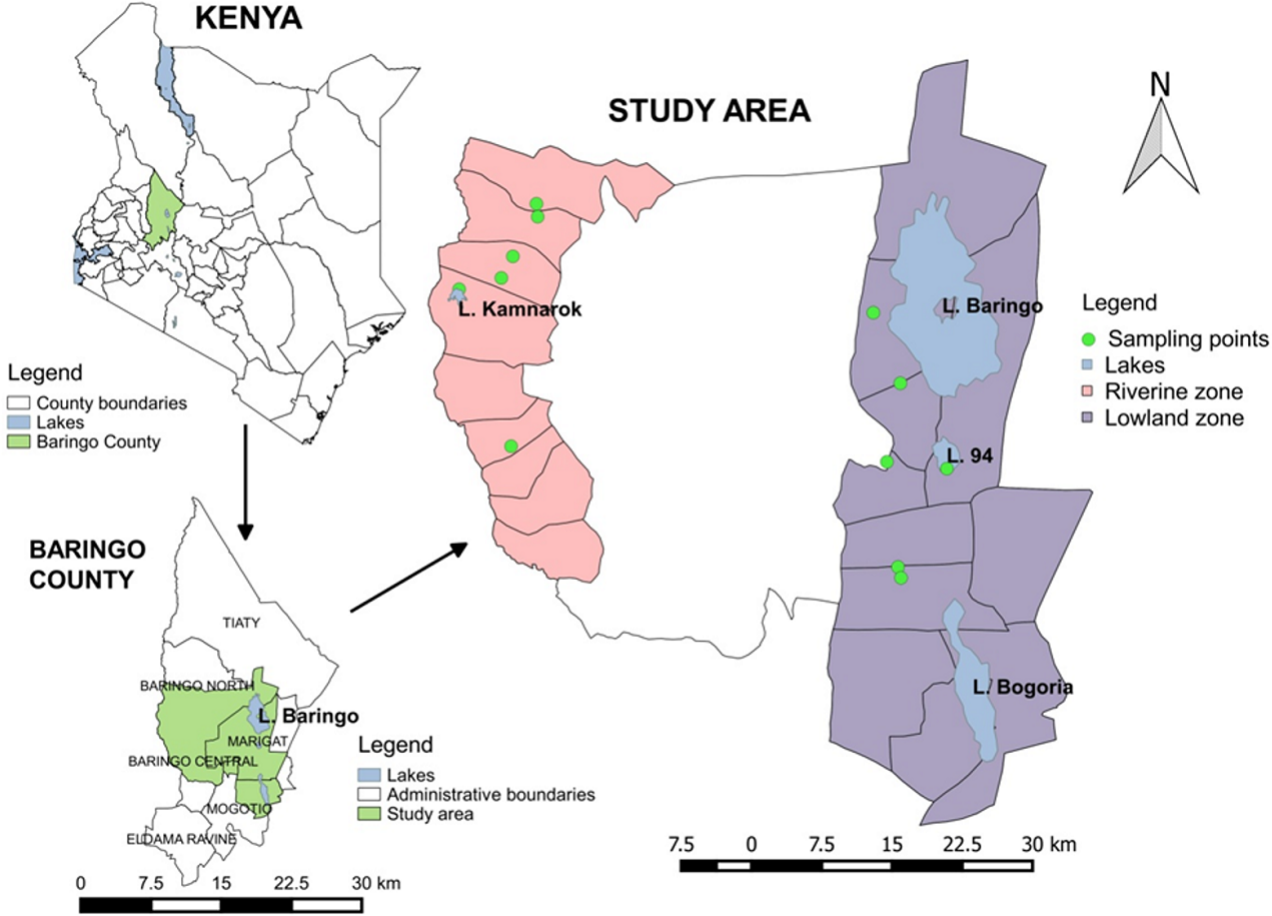
Map of study area showing mosquito sampling points in Baringo County.

The study was conducted in Baringo County of Kenya, lying between longitudes 35.602°E and 36.277°E and latitudes 0.541°N and 0.723°N at altitudes ranging between 870 m and 2499 m above sea level (Fig 1). There are four lakes within the study area, two of which are permanent (Lake Baringo and Lake Bogoria) while the other two are seasonal (Lake 94 and Lake Kamnarok). Most rivers in the area cease to flow during the dry season and are often characterized by pockets of small pools along the riverbed, which provide suitable low level breeding micro-habitats for mosquito vectors throughout the year. The mean annual rainfall is about 650 mm with temperature ranges from 30 ^0^C to 37 ^0^C. The region is mainly an agro-pastoral area and most houses are constructed using different materials reflecting socioeconomic status. The three main communities found in Baringo are Tugen, Pokot and Ilchamus who keep cattle, goats and sheep. While Tugen and Ilchamus grow crops as well as keep animals, the Pokots are purely pastoralists[25]. The Ilchamus community occupies a small area around Lake Baringo while the other two communities occupy Kerio valley and the highlands. Malaria is the major vector borne disease that occurs throughout the county.

### Selection of study zones and sites

Twelve sampling sites were selected across two zones namely: lowland, lying at an elevation of 1,000 m asl and below and riverine zone, lying between 1,100 m and 1,200 m (Fig 1). The two ecological zones were selected based on existence of water bodies such as lakes, rivers, ditches and swamps as possible sources of adult mosquitoes in the houses.

### House type selection and identification

**Fig 2:**
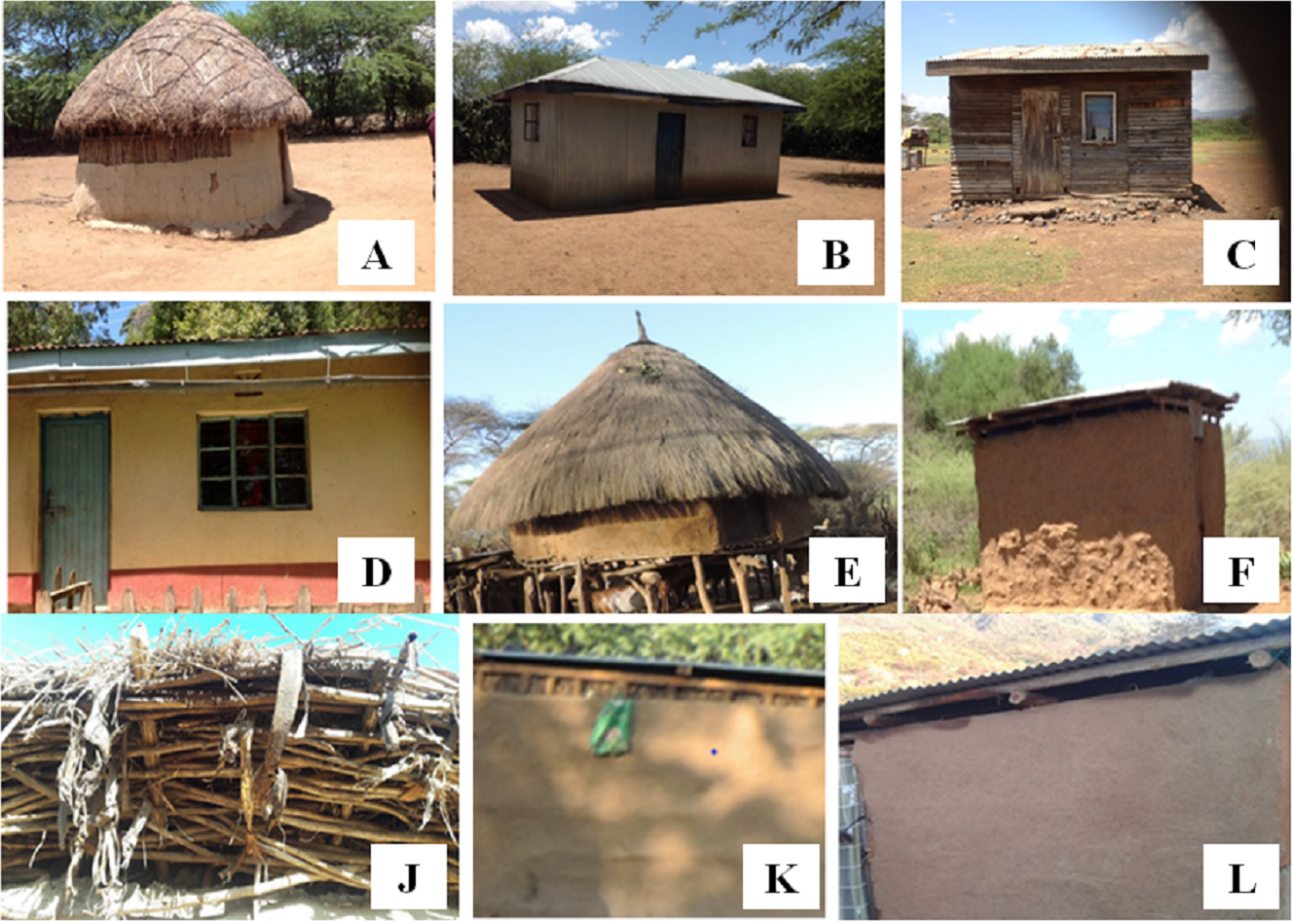
**Examples of house structures that were sampled in Baringo County: Grass-Mud (A), Iron-Iron (B), Iron-Wood (C), Iron-Stone (D), Bororiet (E), Iron-Mud (F). Eave types: Closed Eave Grass-Mud House (J), Closed Eave Iron-Mud House (K), Open Eave Iron-Mud House (L).** Order of naming houses: Roof-Wall.

Six types of houses based on material used for construction of the roof and the wall were selected (Fig 2A–2F). They were identified as; Grass thatched roof-Mud wall (GM), corrugated Iron sheet roof-corrugated Iron sheet wall (II), corrugated Iron sheet roof-Mud wall (IM), corrugated Iron sheet roof-Wooden wall (IW), corrugated Iron sheet roof-Stone wall (IS) and Grass thatched roof-Stone wall (GS). More grass thatched roof-mud walled houses were selected for sampling due to their predominance particularly in the riverine zone. The riverine zone also had unique design of grass thatched roof-mud walled houses raised on stilts (locally known as “bororiet”). The selected houses either had open eaves or closed eaves (Fig 2J, 2K and 2L). Eave is the space between the top of the wall and the overhanging roof. Houses made of corrugated iron sheets both in the roof and wall were the majority and all of them had open eaves. Houses with corrugated iron sheet roofs and mud walls had open eaves and rough walls (Fig 2F). The houses with wooden walls were made of timber which often left spaces in between due to poor construction or wearing out of pieces of timber. The stone walled house with corrugated iron sheet roof was the modern design made of improved materials with plastered walls usually with a finishing of paint (Fig 2D). This type of house did not have a gap between the top of the wall and the roof and was fitted with a ceiling. It also had well fitted doors and windows.

The exact positions of the houses were recorded with a hand-held global positioning system (GPS) receiver (Garmin eTrex 10) and a unique identification number was allocated to each house to avoid confusion during each sampling period as they were sampled repeatedly. The details of each of the houses sampled were recorded and these included the construction material used for the roof and wall, whether the house had open or closed eaves and presence of animal shed nearby. A similar number of each house type could not be sampled because they were not naturally available around aquatic habitats which were used as focal points for house selection. Besides, building standards were not the same due to different social economic status in the natural rural set up. Of the three “bororiet” houses that were available in the riverine zone, one was selected for sampling because it was in the vicinity of other different house types that were targeted for sampling.

### Sampling and identification of mosquitoes

Indoor-resting mosquito vectors were estimated once every month for each of the 74 houses from June 2015 to May 2016. The two zones of the study area were covered during the first week of the month by two entomological teams working simultaneously. Collections were made by pyrethrum spray catch method between 0600 and 0830hrs[26]. Mosquitoes were transported to Division of Vector Borne Diseases laboratory in Marigat (Baringo County) where they were morphologically identified to species level under dissecting microscope using taxonomic keys[27].

### Measurement of meteorological factors

Monthly maximum and minimum air temperatures were sourced from the International Research Institutes (IRI) of climate and Society’s database [28,29] during the study period (June 2015-May 2016). Average temperature for the month was calculated by averaging minimum and maximum temperatures for each day. The monthly average rainfall data used was obtained from University of California Santa Barbara (UCSB) Climate Hazards Group Infra Red Precipitation with Station Data (CHIRPS) v2p0[29].

### Statistical analysis

The relative abundance of malaria vectors in house types was expressed as the percentage of the total number of mosquitoes collected. Generalized Estimating Equation (GEE) model was used whereby the unique house code was entered as the subject variable and the month as the within-subject variable in most of the analyses. The type of model used was Poisson with log linear link and *An*. *gambiae* as the response or dependent variable. *Anopheles gambiae* counts once a month were taken as the repeated measures of a particular house type. House type, eave type, roof type and wall type were used as independent predictors. Based on the sample size per house type, only grass-mud (GM), iron-iron (II), iron-mud (IM) and iron-wood (IW) houses were considered in statistical analysis. Likewise, malaria vectors *Anopheles funestus* and *Anopheles pharoensis* were not included in the GEE analysis because they were few and were not found in all house types.

### Ethical statement

The study involved intrusion of privacy during indoor resting mosquito collections and interruption of house owners’ daily routine. Thus, signed consent was sought from the household head prior to sampling. The investigators involved in mosquito sampling were provided with protective gear which included transparent goggles and gas masks, overalls and gum boots. The study was approved by the Kenyatta National Hospital and University of Nairobi Ethics and Research Committees (KNH-ERC/R/75). It also received ethical clearance from World Health Organization (WHO) protocol ID B20278.

## Results

### Abundance of malaria vectors in different house structures in Baringo County

Three species of malaria vectors namely; *Anopheles funestus*, *Anopheles gambiae* and *Anopheles pharoensis* were identified in different houses in Baringo County. Based on cumulative monthly mosquito collections over a period of 12 months, corrugated Iron sheet roof-Mud walled houses had a higher average number of malaria vectors per house per sample (8.1) followed by Grass thatched roof-Mud walled houses (6.6) (Table 1). Whereas the corrugated Iron sheet roof-Stone walled houses had the least average number of vectors of malaria (0.6), the Grass thatched roof-Stone walled house had a high average number of malaria vectors per sample (64.6). The Grass thatched roof-Stone walled house had both *An*. *gambiae* and *An*. *pharoensis* mosquitoes while corrugated Iron sheet roof-Stone walled houses had *An*. *gambiae* only.

**Table 1:** Cumulative malaria vector abundance in different house structures in Baringo County during the 12-month sampling period.

House types (N)	Vector species abundance				Average/House/Sample
Roof-Wall	*An*. *funestus*	*An*. *gambiae*	*An*. *pharoensis*	Total (%)	
Grass-Mud (23) (GM)	29	1788	4	1821 (36.9)	6.6
Iron-Iron (29) (II)	0	662	1	663 (13.4)	1.9
Iron-Mud (16) (IM)	33	1500	14	1547 (31.3)	8.1
Iron-Wood (3) (IW)	0	104	16	120 (2.4)	3.3
Iron-Stone (2) (IS)	0	14	0	14 (0.3)	0.6

N-Number of houses; %—Percentage of the total vectors collected from each house type; Note: Sampling of each house was done one day of each month

In Kamnarok village within the riverine zone, a house made of grass thatched roof and mud wall (GM) but raised on stilts had lower average number of malaria vectors (5.8 per collection) compared to houses made of same materials about 50 metres away but at ground level (30.5 mosquitoes per house per collection).

### Variations in mosquito abundance in houses with open and closed eaves in Baringo County

Most houses in the lowland and riverine had open eaves (84.5%) compared to closed eaves (15.5%). The average number of malaria vectors per house with open eaves was higher (25.96) than that for houses with closed eaves (4.96). A significant difference was obtained when GEE analysis was performed to compare abundance for *An*. *gambiae* in closed eave houses and open eave houses (Table 2). When all house types were pooled together and *An*. *gambiae* abundance compared between houses with open and closed eaves, the difference was statistically significant (p<0.000). Analysis was done for only grass thatched roof-mud walled houses to compare *An*. *gambiae* abundance between open and closed eaves. The difference was still statistically significant (p = 0.001). The grass thatched roof-mud walled house was the only category which had relatively large numbers of houses with the two eave types for comparison.

**Table 2:** Malaria vector abundance in open and closed eave houses in lowland and riverine zones in Baringo County.

	Variable	Category	Odds Ratio	95%CI	P-value
**All houses**	Eave type	Closed eave	0.131	0.060–0.284	0.000
		Open eave	*		
**GM houses**	Eave type	Closed eave	0.158	0.052–0.480	0.001
		Open eave	*		

GM-Grass thatched roof/Mud wall

*Reference category

### Effect of materials used for construction of roof and wall on vector density in Baringo County

Two types of roof material were recorded among the surveyed houses in Baringo; corrugated iron sheet roofs and grass thatched roofs. Houses in the lowland and riverine zones made of corrugated iron sheet roof were 50 while those made of grass thatch were 24. The average number of mosquitoes per grass thatched roof house was 6.6 while for iron roofed house was 4.8. Analysis by GEE showed that the density of mosquitoes in iron roofed houses was not significantly different from that in grass roofed houses (p = 0.694). On the other hand, four types of materials used to make the walls were recorded namely; corrugated iron sheets, mud, stone and wood (timber). House walls made of corrugated iron sheets were 29, mud walls were 39, stone walls were 2 and wooden walls were 3. The average numbers of mosquitoes per wall type were; 4.11 for corrugated iron sheet wall, 7.19 for mud wall house, 0.58 for stone wall house and 1.10 for wooden wall house. Since houses made of stone wall and wooden wall were very few, analysis was done for mud and iron wall only. Analysis of mosquito density by GEE showed a significant difference between wall types (p<0.000) in the riverine zone only but not in the lowland zone (p = 0.496) or when the two zones were combined (p = 0.495). In the riverine zone, the odds of getting *An*. *gambiae* in iron wall house were less compared to mud wall house (OR = 0.038).

### Association of overall house structure with vector abundance in Baringo County

Statistical analysis was focused on four different house structures which were represented in relatively large numbers in the lowland and riverine zones. Lowland zone and riverine zone were different with regard to *An*. *gambiae* density (13.8 and 5.1 per sample per house respectively) and house type (iron roof-wooden wall houses were only found in lowland while “bororiet” houses were only found in riverine). Thus, all analyses were performed separately for each zone.

Houses in the riverine zone were significantly associated with *An*. *gambiae* abundance (p<0.000) while those in the lowland had no association with *An*. *gambiae* abundance (p = 0.662). In the riverine zone, corrugated iron sheet roof-iron wall houses had significantly lower abundance of *An*. *gambiae* mosquitoes compared to grass thatched roof-mud walled houses which were used as a reference (p<0.000). The odds of finding *An*. *gambiae* in corrugated iron sheet roof-iron wall houses in the riverine zone were less likely than in grass thatched roof-mud walled houses (OR = 0.006, 95%CI = -7.013 to -3.062). When house effects were analyzed while adjusting for rainfall and temperature in the riverine zone, still there was an association between house type and *An*. *gambiae* mosquito abundance in the riverine zone (Table 3). However, these differences were not observed in the lowland zone where *An*. *gambiae* abundance was not significantly different in all house types.

**Table 3:** Effect of house type on mosquito abundance while controlling for rainfall and temperature in the riverine and lowland zones.

Zone	Variable	Category	Odds Ratio	95%CI	P-value
**Riverine**	House type	Iron-mud (IM)	1.533	-1.374; 2.228	0.642
		Iron-iron (II)	0.006	-7.013; -3.062	0.000
		Grass-mud (GM)*			
	Rainfall		1.008	0.005; 0.011	0.000
	Temperature		1.231	-0.017; 0.434	0.071
**Lowland**	House type	Iron-wood (IW)	0.272	-2.826; 0.226	0.095
		Iron-mud (IM)	1.646	-1.287; 2.283	0.584
		Iron-iron (II)	0.762	-2.002; 1.458	0.758
		Grass-mud (GM)*			
	Rainfall		0.989	-0.017; 0.005	0.001
	Temperature		0.856	-0.321; 0.011	0.067

* Reference house

## Discussion

The current study found a higher density of malaria vectors inside houses when the roof was grass thatched and the wall made of mud. This supports several other findings that the economic burden of malaria is highest among the poorest households in the community[30]. The individuals who live in poorly constructed rural houses are exposed to mosquito bites hence increased malaria transmission[31]. Gamage-Mendis *et al*, in their study associated risk of malaria incidence with type of housing construction whereby poorly constructed houses had higher malaria cases[32]. It was observed in Uganda that living in non-mud floors and non-thatched roofs reduced malaria incidence by half compared to living in traditional houses[33]. This is consistent with the findings of a study in Sri Lanka which found more mosquitoes in houses with grass thatched roofs and mud walls compared to those constructed well with plastered walls and tiled roofs[34]. In this study, malaria vector abundance association with different house structures was greater in the riverine zone compared to the lowland zone. This is probably because riverine zone had more varied house designs made of same materials. Of particular interest is the finding that houses built on stilts (locally known as “bororiet”) had relatively fewer malaria vectors than similar houses built on ground level in the riverine zone. Studies conducted in São Tomé, Trinidad and the Dominican Republic also showed that houses raised by stilts had fewer vector mosquitoes than houses built on the ground[31,35]. Incidentally, the “bororiet” house had animals underneath and such animals would have possibly acted as alternative feeding hosts helping to divert mosquitoes from biting human beings, a phenomenon commonly referred to as zooprophylaxis. *Anopheles arabiensis* is the dominant sibling species of *An*. *gambiae* complex responsible for transmission of malaria parasites in Baringo County[36]. The fact that this species is an opportunistic feeder which prefers animal odour than humans could make the houses on stilts with animals underneath a possible control strategy for malaria in the region[37]. In Tanzania, *An*. *arabiensis* mosquitoes were found resting in cattle shed but not found indoors. When animals were absent, this species was found resting indoors and had fed on human blood[38]. This further suggests possibility of protection from *An*. *arabiensis* by zooprophylaxis if animals are kept next to houses.

Wall type had an impact on vector abundance in houses whereby mud walled houses had a higher average number of mosquitoes than other wall types. However, there was no significant difference between roof types even though the mean for grass thatched roofs was higher than mean for corrugated iron sheet roofs. In Burkina Faso, roof type was found to have an effect on *P*. *falciparum* infection, whereby prevalence among children who lived in iron sheet-roofed houses was less than those who lived in mud-roofed houses[20]. This could be because our study investigated vector abundance and there were no houses with mud roofs while that in Burkina Faso investigated *P*. *falciparum* infections. The main types of roofing material in Baringo County are corrugated iron sheets at 60% followed by grass thatched roof at 40%[39]. The proportions of houses sampled in this study were; 66.3% roofs made of iron sheets and 33.7% roofs made of grass thatch. A retrospective analysis of house improvement in Kenya has shown that iron roof houses increased from 1993 to 2009 while grass thatched roofs decreased[24]. There is a general perception that housing characteristics are a function of the household’s socioeconomic situation and have a direct bearing on the health of the occupants[40]. In view of the improved roofing material in Baringo, the greatest challenge is the wall which is predominantly mud.

Differences were observed in vector density between houses with open eaves and those with closed eaves. It was noted that the corrugated iron sheet roof-mud walled houses also had large eaves which could explain why this type of house had unexpectedly higher mean of malaria vectors than grass thatched roof-mud walled houses. It has been demonstrated that malaria vector *An*. *gambiae* mainly enter houses through the eaves [41] and that house design with closed eaves can be a possible malaria intervention[24,42]. Considering the four common houses in the surveyed area of Baringo County, houses with closed eaves had a low mean of mosquitoes compared to houses with open eaves. Research has shown that prevalence of *P*. *falciparum* is higher in children living in houses with open eaves compared to those living in houses with closed eaves[17]. A more recent innovation is the use of insecticide-treated eave tubes which is still under testing and has shown great potential of suppressing mosquito populations [15] with community-wide benefit for houses not fitted with the device[13]. It has also been noted that houses that are ventilated and poorly lit provide ideal indoor resting places for mosquitoes[43]. The findings of the current study attest to this since most mud walled houses which had high abundance of malaria vectors did not have windows to allow light in but had open eaves for ventilation. Similar observations were made in Thailand and Puerto Rico where adult mosquitoes were usually collected in large numbers from some houses but not others[44].

Evidence from investigations done elsewhere has shown that simple modification of housing construction can protect people against mosquito bites[22,33,45]. Atieli *et al*. demonstrated that house design modification by inclusion of a ceiling can reduce mosquito densities considerably[18]. Furthermore, it has been observed that building houses with well fitted windows and doors would reduce mosquito entry as compared to traditional houses[46,47]. In agreement with our study, it is worth noting that the grass thatched roof-stone walled house with open eave and no ceiling had remarkably higher malaria vector density than corrugated iron sheet roof-stone walled house in the same compound. Similar observations have been made in the Gambia and Ethiopia where houses with ceiling, door and window screenings recorded a decrease in indoor resting mosquitoes[48–50]. Malaria vectors *An*. *gambiae* and *An*. *funestus* feed inside houses at night implying that most of the malaria transmission occurs indoors. Therefore, malaria transmission may be affected by house design due to entry rates of the vectors[31]. Generally, modification of house design has been suggested as an intervention for reduction of malaria vectors resting indoors[45,46,48,51].

Other factors that might have contributed to indoor mosquito densities but not studied in this project include wall texture, eave size, distance from breeding sites and the number of occupants per household. Malaria infections and incidence with respect to house types were not investigated under the scope of this study but it is an area that requires further investigation in Baringo County.

## Conclusion

Findings from this study support the association of housing structures with indoor mosquito density. Since malaria vectors mostly bite and rest indoors, house design which hinders mosquito entry and resting should be encouraged as a possible intervention in Baringo County. House modifications such as raising houses on stilts where possible would complement existing protection methods against endophagic and endophilic malaria vectors in Baringo County. Habitat for Humanity (HFH) uses the slogan “Build out Malaria” and recognizes the fact that disease interventions that combine health and housing are essential[43]. Therefore, the innate desire by local communities to upscale their houses could be exploited to support scale-up of integrated vector control even in relatively low-income communities and thus reduce the external donor dependence on malaria control. Due to the low socioeconomic status in the rural villages, stilted houses could easily be achievable among the local communities. The stilted houses were observed mainly in the riverine zone (Kerio valley) among the Tugen community. It is advisable for the Ilchamus community occupying the lowland zone around Lake Baringo to be encouraged to build houses raised above the ground based on the possibility that they can offer some degree of protection from mosquito bites, while animals underneath them can be alternative hosts to humans. The Baringo County Government should, therefore, implement integrated vector management (IVM) strategies including the screening of house eaves in the lowland and riverine zones where malaria vector abundance was high and consider possibility of eave tubes in future.
